# Roles of circRNAs in viral pathogenesis

**DOI:** 10.3389/fcimb.2025.1564258

**Published:** 2025-03-13

**Authors:** Jiayin Liu, Yiming Wang, Meichun Zheng, Jiayuan Du, Mohamed Maarouf, Ji-Long Chen

**Affiliations:** ^1^ Key Laboratory of Animal Pathogen Infection and Immunology of Fujian Province, College of Animal Sciences, Fujian Agriculture and Forestry University, Fuzhou, China; ^2^ Key Laboratory of Fujian-Taiwan Animal Pathogen Biology, College of Animal Sciences, Fujian Agriculture and Forestry University, Fuzhou, China

**Keywords:** CircRNAs, host-virus interaction, virus infection, influenza, oncogenic virus

## Abstract

Circular RNAs (circRNAs) are a class of non-coding RNAs with a covalently closed circular structure, lacking 5’-caps or 3’-poly(A) tails. They are relatively conserved, highly stable, and often exhibit tissue- or cell-specific production in eukaryotic cells. Based on the advances in sequencing technologies and bioinformatics, multiple reports have suggested that viruses and other microorganisms may encode circRNA-like molecules, providing new insights into the physiological and pathological roles of circRNAs. The innate immune system functions as the body’s primary defense mechanism against viral infections. It detects pathogen-associated molecular patterns (PAMPs) and activates signaling pathways to suppress viral replication and limit their spread. CircRNAs are involved in regulation of the host innate immune signaling pathways and play essential roles in viral pathogenesis. It has been shown that circRNAs can regulate gene expression by acting as miRNA sponges or protein sponges, or encoding small proteins in specific cases. For example, previous studies have revealed that circRNAs participate in the host antiviral immune response through the competitive endogenous RNA (ceRNA) network by acting as miRNA sponges. This review highlights research progress in the regulation and functions of host- and virus-encoded circRNAs in host-virus interactions, as well as their potential as diagnostic biomarkers and therapeutic targets in clinical applications.

## 1 Introduction

Circular RNAs (circRNAs) are a unique class of covalently closed circular RNA molecules. Early data initially recognized these cyclic molecules as byproducts of erroneous gene splicing due to limitation of research technologies. In 1976, Sanger et al. reported that plant viroids are a kind of covalently closed single-stranded circular RNA molecules, marking the first identification of circRNAs in nature (Sanger et al., 1976). Recently, circRNAs have been found to be widely present in animals, plants, and some eukaryotic microorganisms and viruses (Wang et al., 2014; Westholm et al., 2014; Broadbent et al., 2015; Lu et al., 2015), and their expression levels are tissue- and stage-specific, varying across different tissues and developmental stages (Memczak et al., 2013).

The circular structure of circRNAs is formed by a process called “back-splicing”, in which 5′ N7-methylguanosine (m7G) cap end and 3′ Poly(A) tail in the pre-mRNA are replaced with a 3′-5′ phosphodiester bond. This distinctive structure makes circRNA highly resistant to RNA nucleases, giving them greater stability than linear RNAs compared to housekeeping genes like GAPDH (Memczak et al., 2013). Back-splicing is carried out by the spliceosome in the nucleus. A single gene can generate different circRNAs through various back-splicing events (Chen, 2020), which may consist of exons, introns, or a combination of both (Jeck and Sharpless, 2014). After back-splicing, the circRNAs formed are transported to the cytoplasm. This entire back-splicing, synthesis, and transport process exists in a dynamic equilibrium state within the cell (Chen, 2020).

In 2011, a hypothesis was proposed, which suggests that RNAs interact with each other through shared microRNA binding sites to form a large regulatory network, referred to as “competitive endogenous RNA” (ceRNA). This hypothesis posits various types of RNA gene expression compete for miRNA binding sites within the ceRNA network. In this model, miRNAs act as regulatory elements, while their downstream mRNAs and proteins encoded by those mRNAs serve as targets for regulation (Salmena et al., 2011).

**Figure 1:**
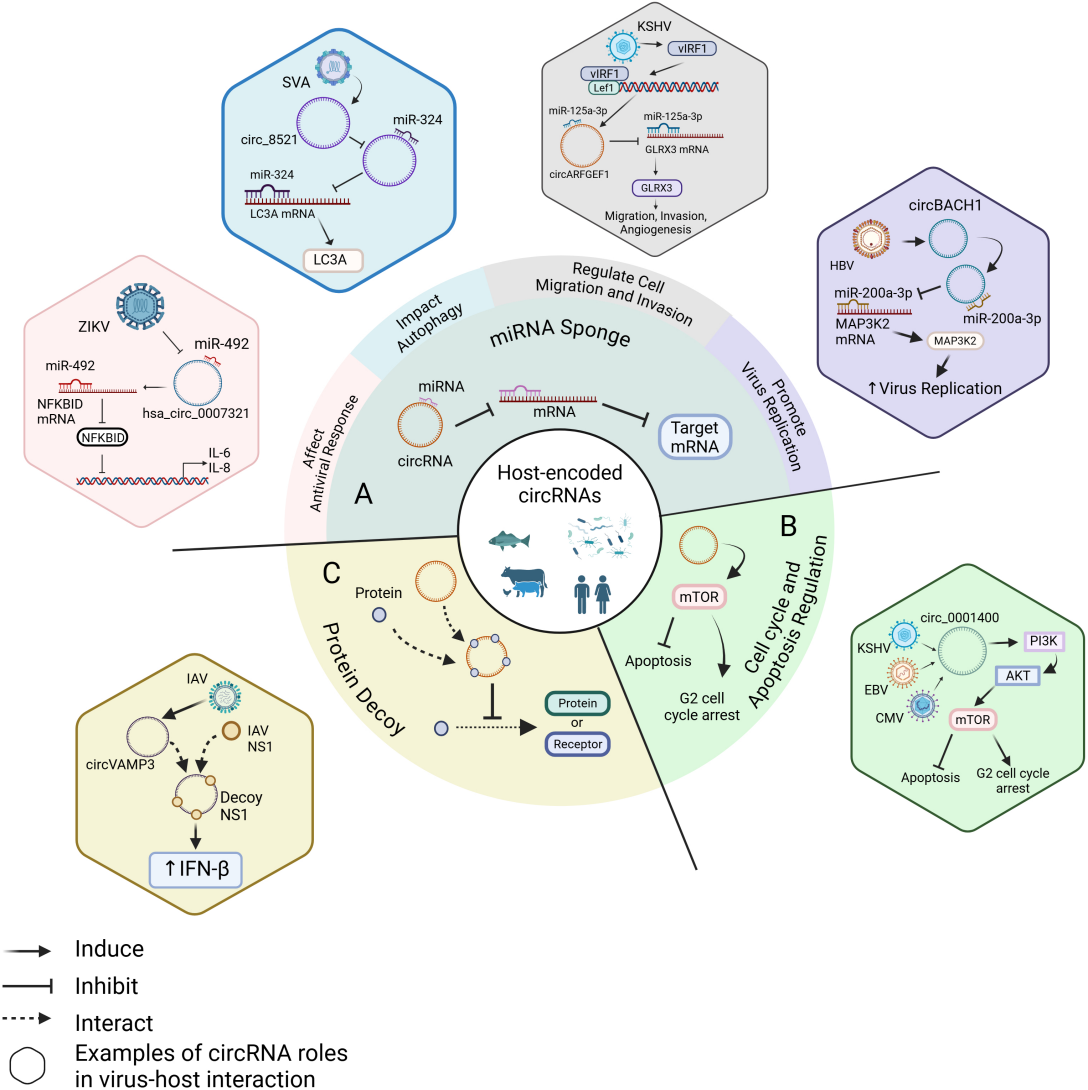
Roles of circRNAs in virus-host interaction. **(A)** As miRNA sponges, host-encoded circRNAs regulate viral infection by affecting antiviral responses, impacting autophagy, modulating cell migration and invasion, and promoting viral replication. For example, ZIKV infection inhibits the binding of hsa_circ_0007321 to miR-492, increasing the availability of miR-492 to bind to NFKBID mRNA. This activates the NF-κB pathway and upregulates IL-6 and IL-8 expression (Kang et al., 2023b). SVA infection upregulates circ_8521, preventing the binding of miR-324 to LC3A mRNA and enhancing LC3A expression (Yang et al., 2024). KSHV induces the expression of vIRF1, which binds to Lef1 and promotes the interaction between circARFGEF1 and miR-125a-3p while reducing the interaction between miR-125a-3p and GLRX3 mRNA. This increases GLRX3 expression, promoting cell migration and invasion (Yao et al., 2021). HBV upregulates circBACH1, which reduces the interaction between miR-200a-3p and MAP3K2 mRNA, leading to increased MAP3K2 expression and enhance HBV replication (Du et al., 2022a). **(B)** KSHV, EBV, and CMV promote circ_0001400 expression, which induces G2 cell cycle arrest and inhibits apoptosis through the PI3K/AKT/mTOR pathway (Tagawa et al., 2023). **(C)** IAV upregulates circVAMP3, which acts as a decoy by binding to NS1, leading to increased IFN-β expression (Min et al., 2023). Created with BioRender.com (accessed on 22 February 2025).

CircRNAs are involved in key physiological processes, including lipid metabolism (Chen et al., 2023b), myocardial and skeletal muscle regeneration (Zheng et al., 2022; Chen et al., 2023a; Zhong et al., 2023), aging and other physiological processes (Ding et al., 2022). They also play a significant role in various diseases, such as heart diseases (Altesha et al., 2019), neurodegenerative disorders (Wu et al., 2023), and eye diseases (Zhang et al., 2020). For instance, circ-Foxo3 can interact with cell cycle-associated proteins, such as cyclin-dependent kinase (CDK) 2, CDK6, and cyclin-dependent kinase inhibitor 1 (p21). CircPABPN has been observed to bind to the human antigen R (HuR). However, the precise molecular mechanisms underlying the interactions between circRNAs and proteins remain to be further elucidated (Du et al., 2016; Abdelmohsen et al., 2017). CircRNAs may function as molecular sponges for miRNAs and proteins, inhibiting their activity (Zeng et al., 2017; Peng et al., 2020). The role of circRNAs as sponges for miRNAs has also been reported in the context of innate immunity against viral pathogenesis and will be discussed with examples later in this review. In addition, some circRNAs have been reported to encode small proteins or regulate the synthesis of rRNA and tRNA (Salmena et al., 2011; Memczak et al., 2013; Zhang et al., 2018). Additionally, it has been documented that circRNAs contribute to innate immune responses to infection with pathogens and regulate viral replication *in vivo* (Chen, 2020). Accumulating evidence has shown that circRNAs within the cells could affect the virulence and pathogenicity of viruses (**Figure 1**). For example, host cell-derived circRNAs can interfere with viral replication, while virus-coded circRNAs can enhance viral infections by promoting immune evasion, affecting viral virulence, and facilitating viral replication (Chasseur et al., 2022; Deng et al., 2022; Ge et al., 2024). Together, these findings provide novel insights into preventing and treating viral infectious diseases. In this review, we introduce the synthesis and biological functions of circRNAs, then discuss their relationship with viral pathogenesis and their potential applications in therapeutic strategies.

## 2 CircRNAs are involved in regulation of host-virus interaction

Viruses invade host cells by attaching to the cellular receptors and utilizing the host biosynthetic machinery for mRNA transcription, protein synthesis and genomic replication. The viral nucleic acids and proteins produced within the host cells are assembled into new virus particles, which are then released to infect nearby cells. Simultaneously, viral infection can trigger a series of host antiviral responses, including activation of the innate immune signaling pathway, induction of autophagy, and the release of interferons to mitigate viral damage to the host. CircRNAs, a recently discovered class of non-coding RNA molecules, have been reported to play critical roles in various processes during viral infection and exhibit diverse functions in viral pathogenesis (**Table 1**).

**Table 1:** List of reported virus-dysregulated host-circRNAs and their functions/mechanisms in viral pathogenesis.

circRNAs	Viruses	Function/Mechanisms	Ref
circVAMP3	IAV	CircVAMP3 interacts with NP and NS1 directly and impairs the transcription and replication of IAV virus genes while reinstating antiviral innate immunity.	(Min et al., 2023)
circRNA Slco3a1	IAV	CircRNA Slco3a1 and circRNA Wdr33 are specifically expressed and closely related to IAV-induced lung injury.	(Wang et al., 2021)
circRNA Wdr33			
circRNA-0050463	IAV	CircRNA-0050463 regulated IAV replication through the circ_0050463/miR-33b-5p/EEF1A1 axis.	(Shi et al., 2021)
circRNA AIVR	IAV	CircRNA AIVR sponged miR-330-3p and regulated the IAV replication by modulating IFN-β production.	(Qu et al., 2021)
circRNA-MerTK	IAV	CircRNA-MerTK regulated the production of IFN-β and modulated the replication of IAV by sponging miR-125a-3p.	(Qiu et al., 2023)
circ-GATAD2A	IAV	Circ-GATAD2A regulated IAV H1N1 virus replication through VPS34-dependent autophagy.	(Yu et al., 2019)
circBACH1	HBV	CircBACH1 promoted HBV replication by regulating the miR-200a-3p/MAP3K2 axis.	(Du et al., 2022a)
circ-ARL3	HBV	Circ-ARL3 enhances HBV replication by sponging miR-1305.	(Rao et al., 2021)
hsa_circ_0001400	KSHV	Hsa_circ_0001400 was found to inhibit KSHV replication, promote cell cycle, inhibit apoptosis, and maintain low immunogenicity of KSHV in cells.	(Tagawa et al., 2023)
circARFGEF1	KSHV	CircARFGEF1 regulates cell migration through the circARFGEF1/miR-125a-3p/GLRX3 axis.	(Yao et al., 2021)
has_circ_0000976	HBV	Diagnostic probes that combine these three circRNAs have been shown to detect HCC accurately.	(Yu et al., 2020)
hsa_circ_0007750			
hsa_circ_0139897			
circSIAE	CVB3	CircSIAE inhibited CVB3 replication via the circSIAE/miR-331-3p/TAOK2 axis, while inhibited the phosphorylation of NF-κB and ERK1/2 proteins,	(Yang et al., 2022a)
hsa_circ_0007321	ZIKV	Hsa_circ_0007321 was found to sponge to miR-492 and thereby regulate ZIKV replication.	(Kang et al., 2023a)
circ_8521	SVA	Circ_8521 enhanced SVA infection by modulating the circ_8521/miR-324/LC3A axis.	(Yang et al., 2024)

### 2.1 Influenza virus

Influenza A virus (IAV) is a negative-strand RNA virus belonging to the influenza virus genus of the *Orthomyxoviridae* family. As a highly infectious zoonotic pathogen, IAV continues to spread among various species, including poultry and mammals, posing a serious threat to the health of humans, livestock, and wildlife. IAV encodes multiple structural and nonstructural proteins, such as hemagglutinin (HA), neuraminidase (NA), nucleoproteins (NPs) and components of the RNA polymerase complex including polymerase basic 1 (PB1) and PB2, and nonstructural protein 1 (NS1). There is increasing evidence that host-derived circRNAs are involved in regulating the viral infection and pathogenesis. For example, circVAMP3 was reported to act as an RNA decoy, directly interacting with the viral NP and NS1. This interaction restricts the transcription and replication of IAV genome (Min et al., 2023).

IAV infection has been linked to lung injury, which, in severe cases, could lead to acute respiratory failure, a leading cause of influenza-related deaths (Ortiz et al., 2013). CircRNA Slco3a1 and circRNA Wdr33 have been identified to be specifically expressed following lung injury caused by IAV. These studies indicate that they are associated with IAV-induced lung injury and may have the potential to become biomarkers for early diagnosis of the lung injury (Wang et al., 2021).

Recently, more host circRNAs have been identified as regulators of IAV pathogenesis by modulating the innate immune responses. For instance, circRNA-0050463 acts as a sponge to miR-33b-5p, promoting the expression of eukaryotic translation elongation factor 1 alpha 1 (EEF1A1) and regulating IAV replication through the circ_0050463/miR-33b-5p/EEF1A1 pathway (Shi et al., 2021). Similarly, circRNA AIVR sponges miR-330-3p and circRNA-MerTK may sponge miR-125a-3p; both circRNAs regulate the IAV replication by modulating interferon β (IFN-β) production (Qu et al., 2021; Qiu et al., 2023). On the other hand, it has been established that IAV infections with several subtypes can induce autophagy in host cells, which facilitates influenza virus replication (Zhang et al., 2019). Interestingly, circ-GATAD2A, a circRNA derived from GATA zinc finger domain containing 2A (GATAD2A), has been found to regulate IAV H1N1 virus replication through vacuolar protein sorting 34 (VPS34)-dependent autophagy (Yu et al., 2019).

### 2.2 Oncogenic virus

In 1910, Rous identified the Rous sarcoma virus (RSV) in chickens and observed the association between viruses and tumorigenesis (Rous, 1910). So far, numerous human and animal viruses have been shown to have capable of inducing cancer. These include Epstein-Barr virus (EBV), hepatitis B virus (HBV), hepatitis C virus (HCV), Kaposi sarcoma-associated herpesvirus (KSHV), human papillomaviruses (HPV), human T-lymphotropic virus 1 (HTLV-1) and human immunodeficiency virus (HIV), as well as various animal retroviruses (Bouvard et al., 2009). While HIV is not directly oncogenic, suppression of the immune system caused by HIV infection increases the risk of cancers linked to oncogenic viruses and immune dysfunction. Among chickens, Marek’s disease virus (MDV), avian leukosis virus (ALV), and reticuloendotheliosis virus (REV) are known to cause cancers (Witter, 1997; Moore and Chang, 2010; Eladwy et al., 2023).

Tumors induced by oncogenic viruses account for a considerable proportion of all tumors. Existing studies have revealed that circRNAs are closely associated with tumorigenesis during the infections by these viruses and exhibit specific expression patterns, providing a valuable reference for disease detection and diagnosis. Previous experiments have demonstrated that circBACH1 promotes HBV replication and liver cancer development by regulating the miR-200a-3p/mitogen-activated protein kinase kinase kinase 2 (MAP3K2) axis, while circ-ARL3 enhances HBV replication by sponging miR-1305 (Rao et al., 2021; Du et al., 2022a). In addition, reports show that hsa_circ_0001400 can inhibit viral replication, promote the cell cycle, and suppress apoptosis in human primary endothelial cells, enabling KSHV to maintain low immunogenicity in cells and evade host immune surveillance. Hsa_circ_0001400 also exhibits an antiviral role in other cell types. For instance, hsa_circ_0001400 can be induced by various pathogens, including KSHV, EBV, and human cytomegalovirus (HCMV) in a variety of cells, suggesting that certain circRNAs may interact specifically with particular viruses in specific cell types (Tagawa et al., 2023). It was thought that KSHV-encoded viral interferon regulatory factor 1 (vIRF1) can promote cell invasion and angiogenesis. It is worth noting that vIRF1 can interact with lymphoid enhancer binding factor 1 (Lef1), activate circARFGEF1 transcription and regulate cell migration through the circARFGEF1/miR-125a-3p/glutaredoxin 3 (GLRX3) axis, contributing to tumor development (Yao et al., 2021).

### 2.3 Other viruses

#### 2.3.1 Coxsackievirus B3

Coxsackievirus B3 (CVB3) is a non-enveloped single-stranded RNA virus belonging to the *Picornaviridae* family and the genus *Enterovirus*. CBV3 is known to cause cardiomyocyte damage, leading to viral myocarditis (Yi et al., 2022). Studies displays that circSIAE inhibits CVB3 replication via the circSIAE/miR-331-3p/thousand and one amino-acid kinase 2 (TAOK2) axis (Yang et al., 2022a). CircSIAE may also affect the phosphorylation of NF-κB and extracellular regulated kinase 1/2 (ERK1/2) proteins, playing a regulatory role in innate immunity (Yang et al., 2022a).

#### 2.3.2 Zika virus

Zika virus (ZIKV) is a single-stranded positive-sense RNA virus belonging to the genus *Flavivirus* within the family *Flaviviridae*. ZIKV is an arbovirus known to cause severe neurological complications (Musso and Gubler, 2016).

Endogenous hsa_circ_0007321 is reported to act as a sponge for miR-492. Upon ZIKV infection, the hsa_circ_0007321 production was suppressed, leading to increased levels of miR-492, which could inhibit the expression of its downstream protein, NF-κB inhibitor delta (IκBδ, NFKBID). This suppression relieves the inhibition of the NF-κB pathway, resulting in the production of proinflammatory cytokines that affect ZIKV infection and replication (Kang et al., 2023a). CircRNAs are also reported to be highly enriched in brain tissue and have been implicated in neurodegenerative diseases (Chen, 2020). Given that ZIKV is known to cause neurological impairment, whether interplay between circRNAs and ZIKV has any effects on the viral pathogenesis in nerve cells remains unclear and is required for further investigation.

#### 2.3.3 Seneca virus A

Seneca virus A (SVA), also known as Seneca Valley virus (SVV), is the sole member of the genus *Senecavirus* in the family *Picornaviridae*. It is a non-enveloped, single-stranded RNA virus. SVA infection causes idiopathic vesicular disease in pigs, leading to vesicular outbreaks in sows and the death of newborn piglets, posing a significant threat to the pork industry (Adams et al., 2015). SVA infection has been shown to cleave the sequestosome 1 (SQSTM1/p62) protein in PK-15 cells, thereby curbing SQSTM1-induced incomplete autophagy and diminishing the autophagy-mediated inhibition of SVA replication (Wen et al., 2021). Recently, circ_8521, a sponge for miR-324, has been reported to promote microtubule associated protein 1 light chain 3 alpha (LC3A) protein expression and autophagy in porcine cells via the circ_8521/miR-324/LC3A pathway, ultimately enhancing SVA infection (Yang et al., 2024).

## 3 Roles of virus-derived circRNAs in viral pathogenesis

Although circRNAs are widely produced in eukaryotic cells, only a small number of viruses have been reported to encode circRNAs so far. These viruses include dsDNA, ssRNA, and retroviruses (Cai et al., 2021). Nearly half of the viral-derived circRNAs range from 200-500 nt in length, with some being longer than half of their viral genome size (Chen et al., 2022). Most viral circRNAs contain short repeat sequences or reverse complementary sequences at their flanks, and some viral-derived circRNAs possess both (Cai et al., 2021). It is speculated that the formation of viral circRNAs may be influenced by the genome size and the presence of specific sequence elements at the flanks (Cai et al., 2021; Chen et al., 2022). Some DNA viruses utilize cellular transcription and RNA splicing pathways, producing viral-derived circular RNA from pre-mRNA (Chasseur et al., 2022). The expression of these viral circRNAs varies across different stages of viral infection (Tagawa et al., 2018). Recently, virus-encoded circRNAs have been observed in different types of viral genomes, including HPV, HBV, and EBV. The viral circRNAs may have bidirectional regulatory functions, modulating both host and viral processes (Zhang et al., 2022). These circRNAs can also regulate the expression of viral genes through the ceRNA network, representing a new type of potential therapeutic target (Yang et al., 2022b).

For instance, MDV, a double-stranded DNA virus of the *Herpesviridae* family, causes tumors in multiple organs in chickens. MDV-encoded circRNA circZMYM3 interacts with several host miRNAs, suggesting a role for the circRNA in MDV-host interaction (Teng et al., 2023). The results also indicate that circZMYM3 is implicated in MDV infection via regulating the host immune response (Teng et al., 2023). Additionally, MDV-encoded circRNAs are closely linked to the main virulence factors of MDV, with the meq and latency-associated transcript (LAT) regions of the MDV genome serving as central hubs for viral circRNA expression during tumorigenesis (Teng et al., 2023). These circRNAs are processed from transcripts associated with virulence and latency, and their expression is stage-dependent (Chasseur et al., 2022).

It has been found that HCMV also encodes various circRNAs, and their expression varies across different cell types. HCMV-encoded circRNAs play critical roles in viral DNA synthesis through the circRNA-miRNA-mRNA network and have been proposed as an excellent diagnostic biomarker for HCMV (Yang et al., 2022b).

CircRNAs encoded by EBV can regulate viral infection, cell cycle, and tumorigenesis. For instance, EBV-encoded circular RNA LMP2A has been associated with distant metastasis and poor tumor prognosis. It may promote tumor angiogenesis via the KH-type splicing regulatory protein (KHSRP)/von Hippel-Lindau (VHL)/Hypoxia-inducible factor-1 α (HIF1α)/vascular endothelial growth factor A (VEGFA) pathway, implying a potential therapeutic target for EBV-associated gastric carcinoma (Du et al., 2022b).

Studies have shown that certain circRNAs encoded by EBV and KSHV exhibit unique stability. For example, EBV expresses circBARTs during all three types of latent infection. CircBART2.2 activates IRF3 and NF-κB by binding to the helicase domain of retinoic acid-inducible gene I (RIG-I). It also promotes programmed cell death-ligand 1 (PD-L1) transcription and facilitates tumor immune escape (Ge et al., 2021). KSHV-encoded circRNAs, such as circvIRF4, are widely expressed in multiple primary effusion lymphoma (PEL) cell lines. These circRNAs can integrate into viral particles after being expressed in host cells, suggesting their involvement in the viral life cycle and potential roles in early infection (Toptan et al., 2018; Abere et al., 2020).

Furthermore, many highly expressed circRNAs are encoded in the replication initiation regions of viral genes. Based on this finding, Ungerleider et al. hypothesized a priming relationship between DNA replication and reverse splicing (Ungerleider et al., 2018, 2019). This phenomenon appears to vary across different viruses. For example, it has been detected that production of atypical viral circRNAs may not rely on the U2 splicing mechanism during the infection of MDV, KSHV, and EBV (Ungerleider et al., 2018; Tagawa et al., 2021). However, the relationship between circRNA formation, DNA replication, and the splicing mechanism of these atypical circRNAs remains to be further investigated.

## 4 Applications of circRNA

As an emerging research hotspot in recent years, circRNAs have been identified as an important class of non-coding RNAs with enhanced stability and lower immunogenicity due to their unique conformation. These properties give circRNAs several advantages over mRNAs in various applications, making them a promising substitute for mRNA in certain areas. For example, RNA cyclization *in vitro* can extend protein expression time in eukaryotic cells (Wesselhoeft et al., 2018). Moreover, circRNAs with specific directed RNA can be generated *in vitro* using cis-ligase ribozyme (RzL). Such circRNA have been shown to express ribonuclease Cas13 *in vitro*, enabling effective antiviral activity against targeted viruses (Su et al., 2024). Such circRNA-based approach significantly enhances RNA editing efficiency and holds great potential as a therapeutic strategy for various diseases.

CircRNAs also exhibit specific expression patterns during cancer development, suggesting that circRNAs may be developed as valuable tools for the diagnosis and prognosis prediction of cancer. Microarray analysis and transcriptome sequencing are widely used to identify differential circRNA expression between healthy individuals and cancer patients, enabling early cancer diagnosis (Zhao et al., 2021). Through microarray and qPCR, researchers identified several circRNAs elevated in the plasma of HBV-related hepatocellular carcinoma (HCC) patients, including has_circ_0000976, hsa_circ_0007750, and hsa_circ_0139897. A diagnostic probe combining these three circRNAs has been shown to detect HCC more accurately than alpha-fetoprotein (Yu et al., 2020). In terms of treatment, circZNF451 activates the E74-like factor 4-Interferon regulatory factor4 (ELF4-IRF4) pathway by complexing with E3 ligase tripartite motif 56 (TRIM56) and RNA‐binding protein Fragile X‐Related Protein 1 (FXR1). This activation induces macrophage polarization and CD8+ T cell exhaustion, reshapes the tumor immune microenvironment, and impacts the effectiveness of anti-programmed death 1 (PD1) therapy (Gao et al., 2022). Besides, circ_0109046, circ_0000437, and hsa_circRNA_079422 were found to be specifically expressed in the development of uterine cancer, suggesting that they may serve as a useful indicator for diagnosing uterine malignancy (Li et al., 2021, 2023; Li and Liu, 2022). In HCC, the expression of hsa_circ_0004018 and hsa_circ_0003570 is downregulated in tumor tissues. Notably, higher expression levels of these circRNAs were reported to negatively correlate with survival rates in HBV-HCC patients, making them a potential prognostic biomarker for HCC (Kang et al., 2023b).

Furthermore, linear mRNA vaccines face challenges such as high production complexity and unstable expression (Niu et al., 2023). CircRNAs, on the other hand, can be rapidly generated *in vitro* without requiring extensive modifications. Their circular structure offers greater stability and more consistent protein expression than mRNAs (Wang et al., 2024). These advantages make circRNAs excellent candidates for vaccine development, either as vaccine candidates themselves or as delivery vectors.

In addition, circRNAs encoding protein-coding antigens have been shown to elicit robust immune responses in mice and exhibit powerful anti-tumor effects (Amaya et al., 2023; Wang et al., 2024). For instance, several circular RNA vaccines for monkeypox virus (MPXV) have been developed. Following two immunizations, these vaccines elicited high titers of MPXV antigen-specific antibodies in immunized mice and induced immune responses comparable to or superior to those induced by linear mRNA vaccines (Zhou et al., 2024). Recent developments in circRNA-based vaccines for severe acute respiratory syndrome coronavirus (SARS-CoV-2) and ZIKV had demonstrated robust protective efficacy, offering novel avenues for the development of circRNA-based vaccine platforms (Qu et al., 2022; Liu et al., 2024). However, the technology for large-scale development and production of circRNA vaccines requires further refinement, and considerable progress is needed before the clinical implementation.

## 5 Summary

Recently, circRNA has attracted wide attention from the scientific community as an emerging research hotspot. They are a key component of the ceRNA network, with some circRNAs able to interact with multiple miRNAs. These miRNAs, in turn, regulate the expression of various target mRNAs, creating an intricate regulatory network that is involved in numerous physiological and pathological processes. Studies have shown that host-derived circRNAs may play a crucial role in viral pathogenesis, especially oncogenic viruses. On the other hand, viruses can also express their circRNAs within the cells, which is closely associated with viral infection and pathogenesis. Examining the relationship between circRNAs and viral pathogenesis can help deepen our understanding of virus-host interaction. However, the precise mechanism by which circRNAs are implicated in many viral infection processes remains unclear and requires further investigation. Importantly, circRNAs hold great potential to be developed as new vaccines, biomarkers, and therapeutic agents. Despite the importance, these applications are still in their early stages and need further exploration. At present, there are many challenges in the study of circRNAs. For example, our understanding of the formation of circRNAs, back-splicing, and their role in the ceRNA network remains elusive. The differential expression levels of circRNAs in different tissues and the interference of their linear mRNA cognate pose challenges for their detection and knockout. Also, many traditional research methods used for ncRNAs are unsuitable for investigation of circRNAs, making it urgent to develop effective and suitable techniques for further studying circRNAs. Furthermore, there is currently no reliable method to generate animal models with specifically edited circRNA genes, which limits *in vivo* studies. Addressing these issues will enable better advancement in circRNA research and its applications.
